# Rapid sequence versus early surgical stabilization of severe chest wall injuries: a propensity score-matched analysis from a multicenter trauma registry

**DOI:** 10.1186/s13054-026-06155-x

**Published:** 2026-06-25

**Authors:** C. Spering, R. Lefering, W. Lehmann, H. Awan Malik, S. Schulz-Drost

**Affiliations:** 1 Department of Trauma Surgery, Orthopaedics and Plastic Surgery, Goettingen University Medical Center, Universitaetsmedizin Goettingen, Robert-Koch-Strasse 40, Göttingen, 37075 Germany; 2 Institute for Research in Operative Medicine (IFOM), University of Witten / Herdecke, Cologne, Germany; 3 Department for Trauma Surgery, Helios Hospital Schwerin, Schwerin, Germany; 4 Department for Trauma- and Orthopedic Surgery, University Hospital Erlangen, Erlangen, Germany; 5 Committee on Emergency Medicine, Intensive Care and Trauma Management (Sektion NIS) of the German Trauma Society (DGU), Berlin, Germany

**Keywords:** Thoracic trauma, Flail chest, Rib fractures, Surgical stabilization, Timing of surgery, Propensity score matching.

## Abstract

**Background:**

The optimal timing for post traumatic Chest Wall Reconstruction (CWR) in severely injured / polytraumatized patients with severe chest wall instability remains a subject of debate. While early surgery within 72 h is associated with improved outcomes, the efficacy and safety of an even earlier “rapid sequence” approach on the day of admission are unclear. This study aims to compare outcomes of severely injured patients undergoing rapid sequence surgery (Day 0) versus early surgery (Days 1–3).

**Methods:**

A retrospective analysis was conducted using data from the TraumaRegister DGU^®^ (2015–2023). Patients with serious chest wall injuries (AIS_Thorax_ ≥3), an Injury Severity Score (ISS) ≥ 9, who survived the first 48 h and underwent CWR were included. Patients were stratified into a “Rapid Sequence” group (surgery on Day 0) and an “Early” group (surgery on Days 1–3). Propensity score matching (PSM) was performed to balance baseline characteristics, including injury patterns, demographics, and initial physiological status. Primary outcome was in-hospital mortality. Secondary outcomes included sepsis, multi-organ failure (MOF), and length of stay.

**Results:**

From an initial cohort of 34,659 patients with severe chest wall injuries, 2,498 operatively treated patients with a known date of surgery were analyzed. 1,168 (46.8%) underwent rapid sequence surgery (Day 0) and 567 (22.7%) underwent early surgery (Days 1–3). Before matching, the Rapid Sequence group had a higher ISS (27.7 vs. 26.0), a higher incidence of severe head trauma (14.4% vs. 9.2%), and significantly higher mortality (8.4% vs. 4.1%). PSM yielded 500 matched pairs. Despite matching, the Rapid Sequence group retained a higher baseline injury burden (mean ISS: 28.1 vs. 26.2; mortality prognosis (Revised Injury Severity Classification, Version III (RISC III) Score): 16.2% vs. 10.7%). The primary outcome showed a nearly threefold higher mortality rate in the Rapid Sequence group (10.6% vs. 3.6%; *p* < .001). Rates of sepsis (14.6% vs. 12.0%) and MOF (33.6% vs. 28.3%) were also higher in the rapid group, though not statistically significant.

**Conclusion:**

In this large registry analysis, rapid sequence CWR on the day of admission identified a distinct subgroup of patients with more severe concomitant injuries and higher baseline risk. The higher mortality in this group likely reflects residual confounding by indication and survivorship bias, rather than a detrimental effect of rapid surgery per se. This suggests that the decision for immediate surgery is likely driven by life-threatening concomitant injuries not fully captured in the matching model, identifying a patient population with an intrinsically higher risk of death. Our findings therefore do not justify a blanket Day-0-for-all strategy, but are consistent with the broader literature suggesting that CWR performed within 72 h is beneficial when timing is individualized to overall injury severity and physiological stability.

## Introduction

Severe chest wall injuries with serial rib fractures or flail chest are among the most important determinants of adverse outcome after blunt trauma. Rib fractures occur in up to 50% of patients with significant thoracic trauma and are associated with increased rates of pneumonia, prolonged mechanical ventilation, and mortality, particularly when multiple ribs are involved or when a flail segment is present [1–3]. Over the last decade, surgical stabilization of rib fractures (SSRF) has evolved from an experimental technique to an evidence-based component of modern chest trauma care. Because the chest wall consists of more than just ribs and the surgical approach involves more than simple rib repair, post-traumatic Chest Wall Reconstruction (CWR) also encompasses the management of muscles, nerves, vessels, and skin, as well as a thorough understanding of chest wall biomechanics [4, 5].

Randomized trials and high-quality meta-analyses have demonstrated that, in patients with flail chest, SSRF reduces pneumonia, need for tracheostomy, duration of mechanical ventilation, and length of ICU and hospital stay compared with non-operative management [1, 6, 7]. These findings have been extended to selected non-flail patterns with multiple displaced fractures [1, 8], and are reflected in consensus guidelines from the Chest Wall Injury Society (CWIS) and, more recently, in a joint position paper from the World Society of Emergency Surgery (WSES) and CWIS [9, 10].

Beyond the question of “whether” to operate, the timing of CWR has emerged as a major determinant of outcome. In a multicenter prospective evaluation, Pieracci et al. showed that for each day of delay in SSRF the odds of pneumonia increased by 31%, of tracheostomy by 26%, and of prolonged ventilation by 27%, leading to the recommendation that SSRF, when indicated, should be performed “as early as possible,” ideally within 72 h of injury [11]. Registry-based analyses and single-center series corroborate that early CWR within 48–72 h is associated with fewer pulmonary complications and shorter ICU and hospital stays compared with delayed fixation [2, 5, 12, 13].

These data have stimulated the hypothesis that an even more aggressive “rapid sequence” approach – CWR on the day of admission (Day 0), often within the first 12 h - might further improve outcomes by immediately restoring chest wall biomechanics, improving ventilation, and attenuating the inflammatory cascade of polytrauma [4, 5, 14]. Rapid stabilization has been shown in single-center series to permit ultra-early extubation, sometimes within hours after surgery, even in severely injured patients [5, 12, 14, 15]. Biomechanical work clearly supports that a flail or imploding chest wall segment causes paradoxical motion, impaired lung inflation, and increased spinal instability, and that rigid plate fixation restores near-physiologic mechanics [4].

However, the cohort selected for immediate “Day 0” surgery in routine practice likely differs systematically from those stabilized on Days 1–3. In real-world trauma systems, the decision to proceed to emergency thoracotomy, hemostatic laparotomy, or combined procedures with concomitant CWR is driven by life-threatening injuries and hemodynamic instability. In addition, the most severely injured polytrauma patients often present with profound shock, coagulopathy, and severe pulmonary contusion - conditions that may simultaneously mandate and complicate immediate CWR. Thus, any comparison of outcomes by timing of CWR is potentially confounded by indication and survival bias.

To date, no large-scale, multicenter analysis has specifically compared “rapid sequence” Day 0 CWR with “early” CWR on Days 1–3 in a modern trauma system, after accounting for baseline differences in injury pattern, physiology, and care pathways. Using the TraumaRegister DGU^®^ (TR-DGU) of the German Trauma Society, we sought to address this gap. We hypothesized - based on the extrapolation from early versus late data - that Day-0-CWR would be associated with lower in-hospital mortality and fewer complications than CWR performed on Days 1–3.

The primary objective of this study was to compare in-hospital mortality between polytrauma patients with severe chest wall injury undergoing rapid sequence CWR on Day 0 versus early CWR on Days 1–3, using propensity score matching to balance key prognostic variables. Secondary objectives were to compare the rates of sepsis and multi-organ failure (MOF), duration of mechanical ventilation, and ICU and hospital length of stay between the two groups.

## Methods

### Study design and data source

We performed a retrospective cohort study using prospectively collected data from the TraumaRegister DGU^®^ (TR-DGU) of the German Trauma Society (Deutsche Gesellschaft für Unfallchirurgie, DGU).

The TR-DGU was founded in 1993. The aim of this multicenter database is the pseudonymized and standardized documentation of severely injured patients.

Data are collected prospectively in four consecutive phases: (A) prehospital phase, (B) resuscitation room and initial surgery, (C) intensive care unit, and (D) discharge. Documentation includes detailed information on demographics, injury patterns, comorbidities, prehospital and clinical management, intensive care unit (ICU) course, key laboratory findings including transfusion data, and outcome. Inclusion criteria are admission to the hospital via the trauma room with subsequent intensive or intermediate care (IMC) monitoring, or arrival to the hospital with vital signs and death before admission to the ICU.

The infrastructure for documentation, data management and data analysis are provided by the AUC - Academy of Trauma surgery (AUC - Akademie der Unfallchirurgie GmbH), which is affiliated with the German Trauma Society. The scientific management of the registry is the responsibility of the Committee on Emergency Medicine, Intensive Care and Trauma Management (Sektion NIS) of the German Trauma Society. Participating hospitals enter their pseudonymized data into a central database via a web-based application. Scientific evaluations are approved after a review process by the Section NIS.

Participating hospitals are primarily located in Germany (90%), but an increasing number of clinics from other countries are also contributing data (currently from Austria, Belgium, Finland, Luxembourg, Slovenia, Switzerland, the Netherlands, and the United Arab Emirates). Currently, approximately 30,000 cases from nearly 700 hospitals are entered into the database each year. Participation in TR-DGU is voluntary. For the clinics belonging to the German network of trauma care (TraumaNetzwerk DGU^®^), at least the entry of a basic data set for quality assurance is mandatory.

Data used in this analysis were extracted from the 2015–2023 dataset under project ID TR-DGU 2024-018. The study was approved by the scientific committee of the TR-DGU and conducted in accordance with the Declaration of Helsinki. All data were blinded for date, time, and hospital; data were routinely collected for the purpose of quality control, and patients gave their informed consent for the documentation.

### Patient selection

**Fig. 1:**
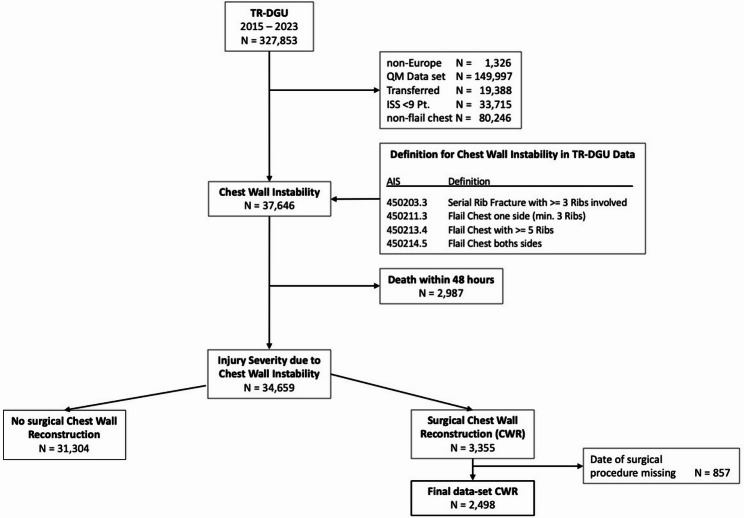
Flow chart of inclusion and exclusion criteria and definition of chest wall instability (CWI). To exclude patients whose injury severity was too high for survival or whose death was not attributable to chest wall instability, all patients who died within 48 h were removed from the final dataset. Of the 34,659 patients meeting the inclusion criteria, 9.7% underwent surgical chest wall reconstruction (CWR), while 31,304 did not. The final dataset for further analysis comprised *N* = 2,498 patients with complete required data

We included (Fig. 1) all patients in the TR-DGU between January 1, 2015, and December 31, 2023, who met the following criteria:

1. Primary admission to a participating hospital in Europe.
2. Age ≥ 16 years.
3. Injury Severity Score (ISS) ≥ 9.
4. Serious chest wall injury, defined as: – serial rib fractures involving ≥ 3 ribs (Abbreviated Injury Scale (2005), AIS 450203.3), or – flail chest (unilateral or bilateral; AIS 450211.3, 450213.4, 450214.5); (AAAM - Association for the Advancement of Automotive Medicine (2008). Abbreviated Injury Scale 2005, Update 2008. Barrington, IL, USA.)
5. Survival for at least 48 h post-admission, to exclude early deaths unrelated to CWR timing.
6. Documented surgical CWR, coded as rib fixation procedures in the registry.
7. Valid calendar date of the CWR procedure, allowing classification by day relative to admission.

We excluded (Fig. 1):

1. secondary transfers from other hospitals.
2. reduced basic documentation only (for quality management).
3. cases with missing CWR date.

To exclude early deaths that are unlikely to be influenced by the timing of CWR and to reduce extreme survivorship bias, we excluded all patients who died within the first 48 h after admission. We also excluded 857 operative cases (25.5% of all CWR patients) with missing surgery date, because a valid timing category could not be assigned. These exclusions may introduce selection bias; however, date imputation was not feasible for a robust timing analysis.

### Exposure and group definitions

The exposure of interest was the timing of CWR, defined by the day of surgery relative to the day of admission (Day 0). Based on prior internal analyses and the clinical timing literature [1, 2, 11], we categorized patients into four groups:

1. Rapid sequence CWR (“Rapid”): Day 0 (day of admission).
2. Early CWR (“Early”): Days 1–3.
3. Delayed CWR: Days 4–6.
4. Late CWR: Day 7 or later.

The primary comparative analysis focused on Rapid versus Early CWR, as these two groups most closely reflect the clinically relevant “rapid sequence” versus “early” strategies within the generally recommended < 72-h window. Patients in the Delayed and Late groups were described descriptively but not included in the primary matched analysis, because their survival beyond 3 days introduces substantial survivorship bias and reflects a primarily conservative initial strategy. In TR-DGU, the timing of CWR is recorded as calendar date but not as exact time for rib fixation procedures. Consequently, a small number of patients may have similar elapsed times from admission to surgery but fall into different day categories (e.g., surgery at 23:00 on Day 0 vs. 02:00 on Day 1). This introduces potential misclassification at the boundary between Day 0 and Day 1, although such situations are expected to be infrequent and unlikely to account for the substantial outcome differences observed between groups.

### Outcomes

The primary outcome was all-cause in-hospital mortality. Secondary outcomes included:

- Sepsis, as defined in the TR-DGU (clinically diagnosed sepsis with organ dysfunction).
- Multi-organ failure (MOF), defined as failure of ≥ 2 organ systems.
- Duration of invasive mechanical ventilation (days).
- Length of stay (LOS) in the ICU (days).
- Total hospital LOS (days).

### Covariates and risk adjustment

Baseline variables considered for adjustment were selected a priori by a multidisciplinary team of trauma surgeons and statisticians, based on their known or plausible association with both CWR timing and outcomes, and informed by CWIS/WSES guidance on SSRF [10, 22]. All clinically plausible variables available in TR-DGU that could influence the decision to operate on Day 0 versus Days 1–3 were considered. A liberal inclusion criterion (*p* < .15) in the propensity score model was used to avoid omitting relevant predictors, with final variable selection additionally guided by clinical plausibility. Composite prognostic scores such as RISC III were not included as covariates in the propensity model in order to avoid potential collider bias; instead, they were used descriptively to characterize baseline risk.

These included:

- Demographics: age (continuous), sex.
- Injury severity: ISS (continuous), AIS_Thorax_, AIS_Head_, AIS_Abdomen_, AIS_Extremities_.
- Presence of polytrauma according to the Berlin definition [16].
- Presence of isolated thoracic trauma (AIS_Thorax_ ≥3 with AIS ≤ 2 in other regions) versus multi-region injury.
- Presence of penetrating injury.
- Thoracic injury patterns: lung contusion, lung laceration, hemothorax.
- Physiological status and early resuscitation: base excess ≤ − 6 mmol/L, coagulopathy, hemoglobin < 7 g/dL, packed red blood cell transfusion in the emergency room or operating room, need for another emergency operation from a predefined list (thoracotomy, laparotomy, pelvic stabilization, emergency neurosurgical procedure, etc.)
- Hospital characteristics: treatment in a Level 1 trauma center.
- Admission characteristics: admission at night (6pm – 6 am), admission on a weekend (Friday 6 pm to Monday 6 am).
- Predicted mortality according to the RISC III score.

Physiological variables (base excess, hemoglobin, coagulopathy, early packed red blood cell transfusion) were limited to those routinely collected at admission and during initial resuscitation. The registry does not capture continuous hemodynamic trajectories or repeated measurements during the first hours, which are likely relevant for the timing decision, and these unmeasured factors cannot be accounted for in the model.

### Propensity score model and matching

To mitigate confounding by indication, we constructed a propensity score model estimating the probability of receiving Rapid sequence (Day 0) CWR versus Early CWR (Days 1–3). We restricted the derivation cohort to patients operated on Day 0–3 (Rapid + Early). A multivariable logistic regression model was fitted with the dependent variable “Rapid (Day 0) CWR” and the covariates listed above. Variables were retained in the final model if they met a liberal inclusion criterion (*p* < .15) and improved overall model fit, guided by clinical plausibility. Several alternative propensity score model specifications, including models incorporating ISS and RISC III, were explored. These variants modestly changed the matched sample but did not eliminate residual differences in ISS, severe head injury, and predicted mortality. Models including RISC III as a covariate markedly reduced the number of matched pairs without resolving the underlying indication bias. The final model thus reflects a pragmatic compromise between covariate balance and sample size. Model discrimination was modest (Nagelkerke’s R² = 0.115), consistent with substantial variation in timing decisions driven by unmeasured factors (e.g., detailed fracture morphology, intraoperative findings, and real-time hemodynamic trajectories).

The final propensity score model included:

- AIS_Thorax_.
- Isolated thoracic trauma vs. combination trauma.
- Presence of relevant injuries outside the thorax (AIS ≥ 3 in other regions).
- Penetrating mechanism.
- Level 1 center.
- Age.
- Sex.
- Packed red blood cell transfusion in trauma room/OR.
- Weekend admission.
- Night-time admission.
- Presence of other thoracic injuries (lung contusion, laceration, hemothorax).
- “Relatively less severe” overall injury pattern (maximum AIS = 3 vs. > 3).
- Need for another emergency operation.

We then performed an exact matching of the rounded percentages (W’keit for Rapid CWR), using a caliper of 0.2 SD of the logit of the propensity score. The matching was restricted to Rapid and Early patients (Day 0 vs. Days 1–3). Balance was assessed by standardized mean differences (SMDs), with values < 0.1 considered indicative of good balance.

### Statistical analysis

Continuous variables are presented as mean ± standard deviation (SD) if normally distributed or median with interquartile range (IQR) otherwise. Categorical variables are presented as counts and percentages. In the unmatched cohort, group comparisons were conducted with Student’s t-test or Wilcoxon rank-sum test for continuous variables and χ² test or Fisher’s exact test for categorical variables.

Within the matched cohort, paired analyses were performed. A two-sided p-value < 0.05 was considered statistically significant. All analyses were conducted using IBM SPSS Statistics (version 29, IBM Inc., Armonk, N.Y., USA).

Missing data for key predictors (age, sex, ISS, AIS region scores, admission time, mortality) were rare and cases with missing values were excluded from propensity score estimation and matching. For selected binary variables with limited missingness (e.g., penetrating injury), missing values were treated as ‘no’ when there was no evidence for systematic under-reporting. We did not perform multiple imputation, because the primary objective was to construct a propensity score suitable for balancing groups within the observed dataset rather than to develop a generalizable prediction model.

## Results

### Cohort description

Between 2015 and 2023, 117,892 patients with ISS ≥ 9 and available data set for this analysis were recorded in the TR-DGU. Of these, 37,646 (32.0%) had severe rib fractures or flail chest and ISS ≥ 9. After excluding patients who died within the first 48 h (*n* = 2,987) to exclude those who died due to more severe concomitant injuries - i.e. severey TBI -, 34,659 patients with severe chest wall injury remained (Fig. 1). Among these, 3,355 (9.7%) underwent operative rib stabilization. This proportion increased with chest injury severity, from approximately 7% at AIS_Thorax_ 3 to 22% at AIS_Thorax_ 4–5. The proportion of cases involving surgical CWR has remained stable over the years (between 9 and 11% of the patients with AIS_Thorax_ ≥3).

**Fig. 2:**
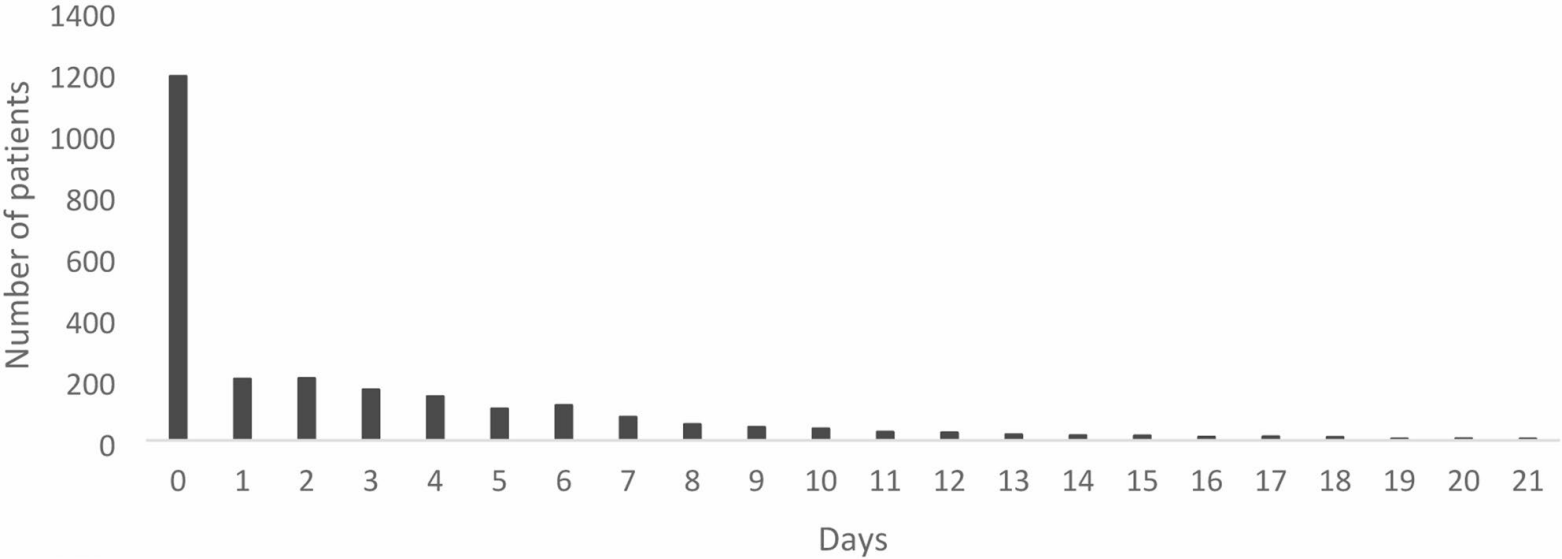
Distribution of Patients with surgical CWR by day. 47.5% received CWR on day 0, 8.0–6.6% on days 1–3

After exclusion of 857 operative cases (25.5%) with missing date of surgical CWR, 2,498 patients of 223 trauma centers remained for timing analysis. The distribution of CWR timing within the groups is schown in Table 1 and the total number of surgically treated patients with CWR by day is illustrated in Fig. 2.

**Table 1:** Distribution of surgical CWR-Patients (*n* = 3,355) within in the study groups

Group	Number of patients	Proportion of all surgical CWR (*n* = 3,355)
**Rapid** (Day 0)	1,168	46.8%
**Early** (Days 1–3)	567	22.7%
**Delayed** (Days 4–6)	360	14.4%
**Late** (Day 7+)	385	15.4%

**Fig. 3:**
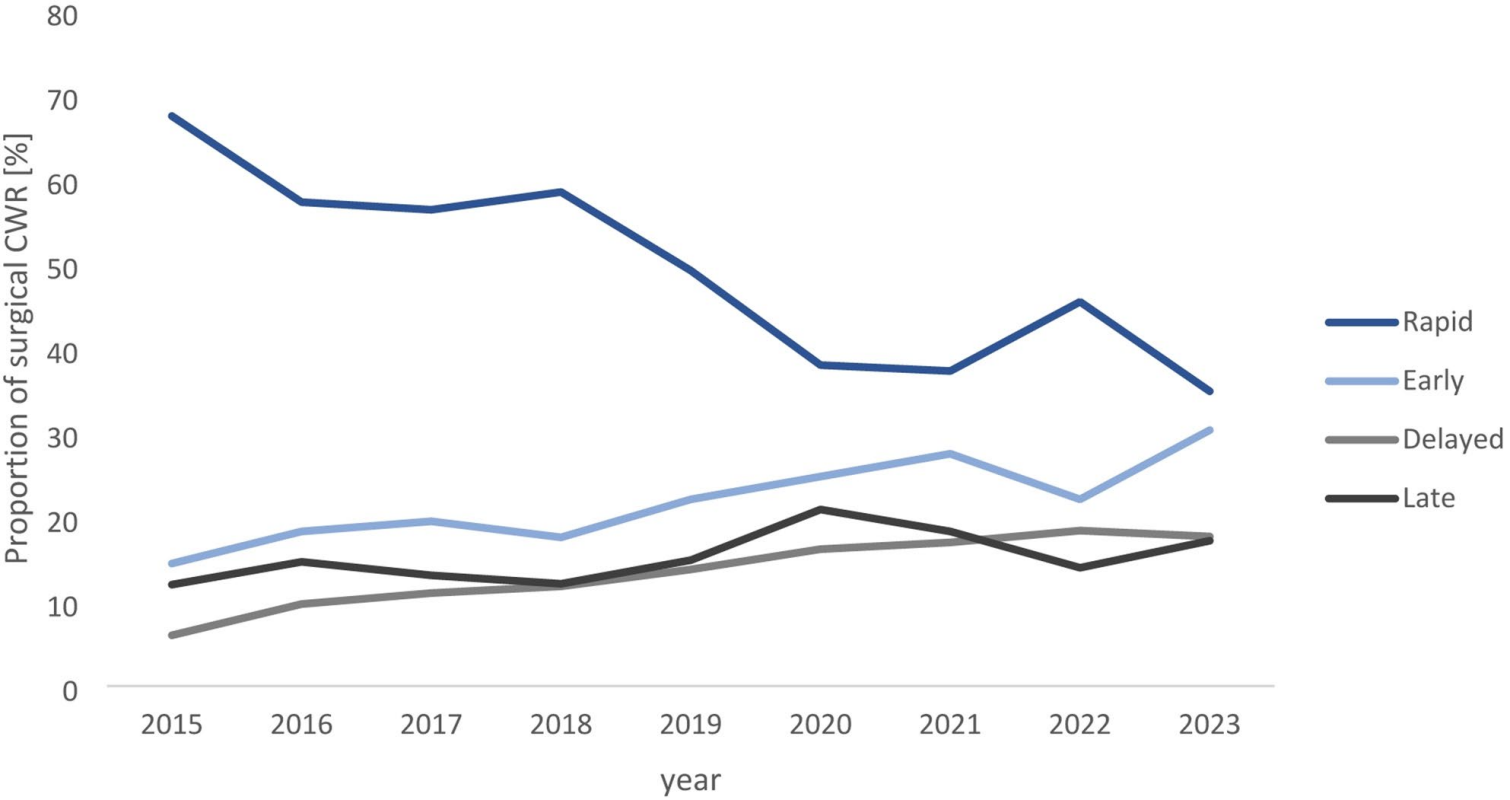
Distribution of the proportion of operatively treated patients with chest wall reconstruction (CWR) across the four timing groups (Rapid: day 0; Early: days 1–3; Delayed: days 4–6; Late: day 7 or later) between 2015 and 2023. There is a marked decrease in the Rapid-CWR group from 67.5% in 2015 to 34.9% in 2023, as well as an increase in the Early group from 14.5% in 2015 to 30.3% in 2023

The proportion of Day 0 CWR decreased modestly over the study period (from 67.5% in 2015 to 34.9% in 2023), while the proportions of Early and later CWR increased (Early group from 14.5% in 2015 to 30.3% in 2023), reflecting a gradual shift from a “rapid sequence” or „delayed“ and „late“ towards a more “early” (1–3 days) strategy in contemporary practice, in line with emerging guideline recommendations (Fig. 3).

### Unmatched comparison: Rapid vs. Early CWR

Before propensity matching, patients in the Rapid group (Day 0; *n* = 1,168) and Early group (Days 1–3; *n* = 567) differed in several clinically relevant characteristics (Table 2):

**Table 2:** Comparison of patients treated rapid (day 0) versus Early (day 1–3) versus delayed (day 4–6) versus late (days 7+)

Attribute	Rapid (day 0) *N* = 1168	Early (day 1–3) *N* = 567	Delayed (day 4–6) *N* = 360	Late (day 7+) *N* = 385
Age	55.7 (18.3)	58.7 (16.5)	59.1 (14.8)	60.8 (15.5)
Age over 80 y.	122 (10.3%)	63 (11.1%)	29 (8.1%)	51 (13.2%)
Gender (male)	902 (76.1%)	447 (78.8%)	271 (75.3%)	306 (79.5%)
ISS	27.7 (13.1)	26.0 (11.8)	26.3 (11.8)	29.4 (13.0)
Severe TBI (AIS 4–6)	171 (14.4%)	52 (9.2%)	36 (10.0%)	50 (13.0%)
Abdominal trauma (AIS 3+)	182 (15.3%)	78 (13.8%)	46 (12.8%)	72 (18.7%)
Trauma to extremities (AIS 3+)	365 (30.8%)	121 (21.3%)	80 (22.2%)	100 (26.0%)
Polytrauma (Berlin)	401 (33.8%)	142 (25.0%)	98 (27.2%)	146 (37.9%)
Penetrating trauma	28 (2.4%)	4 (0.7%)	2 (0.6%)	5 (1.3%)
Sever Rib fractures (AIS)3 4 5	708 (59.7%) 272 (22.9%) 206 (17.4%)	295 (52.0%) 179 (31.6%) 93 (16.4%)	172 (47.8%) 124 (34.4%) 64 (17.8%)	162 (42.1%) 149 (38.7%) 74 (19.2%)
Lung laceration	88 (7.4%)	66 (11.6%)	37 (10.3%)	35 (9.1%)
Hemothorax	368 (31.0%)	261 (46.0%)	174 (48.3%)	185 (48.1%)
Lung contusion	431 (34.8%)	236 (41.6%)	150 (41.7%)	165 (42.9%)
Lung contusion identified after ICU admission	9 (0.8%)	--	2 (0.6%)	2 (0.5%)
Admission at night	373 (31.5%)	189 (33.3%)	106 (29.4%)	124 (32.2%)
Admission during the weekend	543 (45.8%)	228 (40.2%)	208 (57.8%)	151 (39.2%)
TC Level 1 (versus 2–3)	1011 (85.2%)	483 (85.2%)	305 (84.7%)	341 (88.6%)
Transport by helicopter	343 (30.0%)	158 (28.8%)	101 (29.1%)	118 (31.8%)
Emergeny surgical procedure	476 (40.1%)	134 (23.6%)	84 (23.3%)	116 (30.1%)
Blood transfusion (RBC) in TRU/OR	248 (20.9%)	90 (15.9%)	57 (15.8%)	91 (23.6%)
Base Excess <= -6	207 (18.9)	83 (16.2)	53 (16.4)	92 (26.4)
Coagulopathy	176 (15.5)	77 (14.1)	44 (12.7)	80 (21.9)
Hb < 7	23 (1.9)	4 (0.7)	4 (1.1)	10 (2.6)
Sepsis	138 (13.6%)	55 (11.4%)	45 (14.6%)	72 (21.2%)
Organ failure lungs	255 (24.6%)	112 (22.6%)	91 (29.4%)	117 (34.4%)
MOF	331 (31.9%)	136 (27.4%)	88 (28.4%)	136 (39.8%)
Respirator dependency (days)	1 (0–9) MW 6.5	1 (0–9) MW 6.0	1 (0–9) MW 6.2	3 (0–19) MW 10.9
Length of stay ICU	7 (2–17) MW 11.6	8 (3–16) MW 11.5	9 (3–16) MW 12.2	15 (4–27) MW 19.3
Length of stay hospital	18 (10–29) MW 22.9	17 (11–26) MW 22.7	20 (14–30) MW 24.2	27 (20–42) MW 34.4
RISC III Prognosis*	14.6%	11.3%	11.9%	16.5%
Mortality	100 (8.4%)	23 (4.1%)	14 (3.9%)	22 (5.7%)

^*^Significance was defined as p<0.05

Age was slightly lower in the Rapid group vs. Early group (55.7 ± 18.3 vs. 58.7 ± 16.5 years, *p*<.001). The overall injury severity was higher in the Rapid group (ISS 27.7 ± 13.1 vs. 26.0 ± 11.8, *p*=.012). Severe head injury (AIS_Head_ 4–6) was more common in the Rapid group (14.4% vs. 9.2%, *p*<.001), while severe extremity injury (AIS_Extremities_ ≥3) and polytrauma according to the Berlin definition [16] were more frequent in the Rapid group (30.8% vs. 21.3% and 33.8% vs. 25.0%, respectively, both *p*<.001). Penetrating mechanisms were more prevalent in the Rapid group (2.4% vs. 0.7%, *p*<.013) and a greater proportion of Rapid group patients required at least one additional emergency operation (40.1% vs. 23.6%, *p*<.001) and received early blood transfusions (20.9% vs. 15.9%, *p*=.014). The distribution of chest wall injury severity was slightly shifted (*p*<.001), with a higher proportion of AIS_Thorax_ 3 in the Rapid group vs. Early group (59.7% vs. 52.0%) and somewhat fewer AIS 4 injuries (22.9% vs. 31.6%), though AIS 5 injuries are comparable (17.4% vs. 16.4%).

#### Thoracic organ injuries exhibited nuanced differences

As shown in Table 2 lung lacerations were less frequent in Rapid than Early (7.4% vs. 11.6%, *p*=.004). And so were hemothorax and lung contusion somewhat less common in Rapid than Early (31.0% vs. 46.0%, *p*<.001, and 34.8% vs. 41.6%, *p*=.006, respectively), consistent with Rapid CWR decisions often being driven by life-threatening (intra-)thoracic injuries rather than by parenchymal lung pathology.

Reflecting this higher overall injury burden and greater need for emergent intervention, unadjusted mortality was significantly higher in the Rapid group (8.4% vs. 4.1%; *p*<.001). Sepsis and MOF rates were also somewhat higher in Rapid vs. Early (13.6% vs. 11.4%, *p*=.228, and 31.9% vs. 27.4%, *p*=.075, respectively), though differences in ICU / hospital LOS and ventilator days were modest (Table 2).

### Propensity score model and matched cohort

**Fig. 4:**
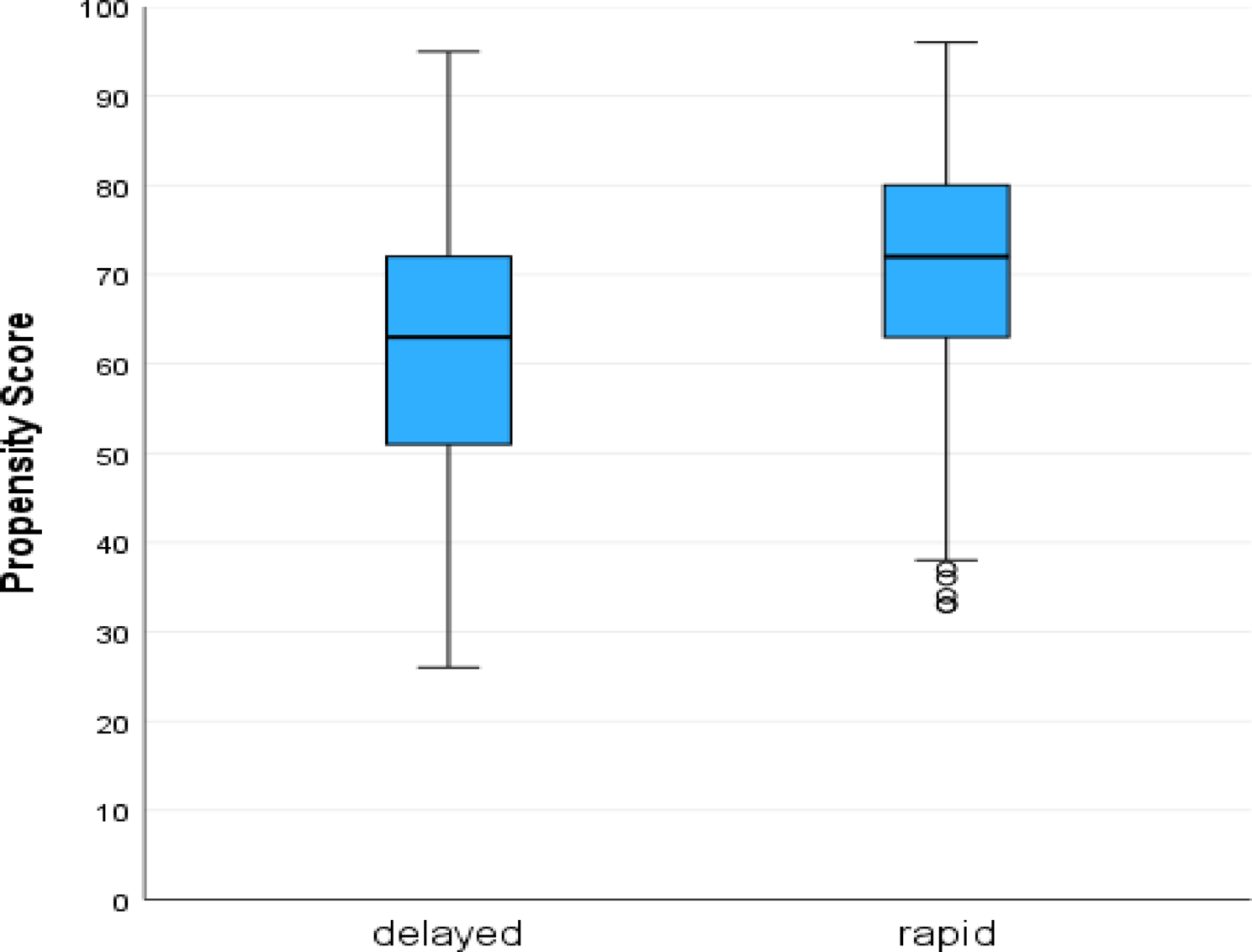
Determinants of rapid sequence surgery and construction of the propensity score–matched cohort. A logistic regression model was used to estimate the probability (propensity score) of receiving rapid sequence surgical stabilization of rib fractures on day 0 versus delayed surgery on days 1–3. Factors associated with an increased likelihood of rapid sequence surgery included relevant extra-thoracic injuries (OR 1.56), penetrating trauma (OR 2.95), admission on a weekend, admission during daytime (cases admitted at night after 18:00 were classified as “rapid” only if operated within 6 h), a lower overall injury burden (maximum AIS = 3 in any body region; OR 1.71), and the need for another emergency operation (OR 2.09). Factors associated with a decreased likelihood of rapid sequence surgery were the presence of additional thoracic injuries (hemothorax, lung contusion, lung laceration), higher age (OR per year increase), and male sex. The model’s explanatory power was modest (Nagelkerke’s R² = 0.115), indicating limited overall ability to predict rapid versus delayed surgery, as shown in the box plot. The resulting propensity score (rounded predicted probability of rapid surgery) was used for exact 1:1 matching, yielding 500 matched pairs (*n* = 1000), each consisting of one patient treated on day 0 (rapid sequence) and one patient treated on days 1–3 (early/delayed)

**Fig. 5:**
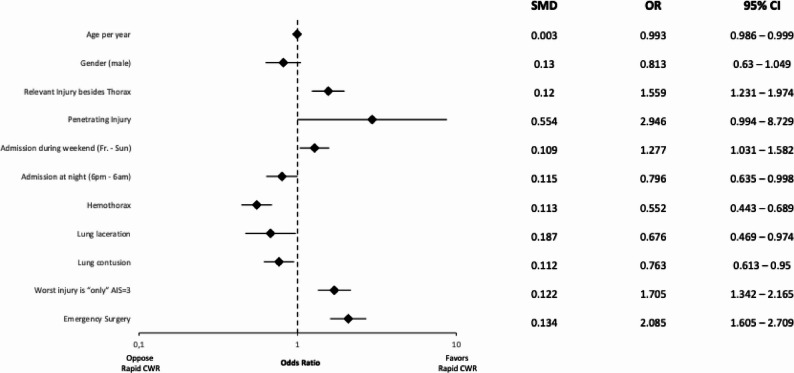
Odds Ratio shown as Forest Plot. An OR > 1 favors rapid CWR, an OR < opposes rapid CWR. The propensity score model for predicting Rapid (Day 0) versus Early (Day 1–3) CWR had modest discrimination (Nagelkerke’s R²=0.115). Shown are the factors that significantly favored Rapid CWR included: relevant injury besides the thorax (OR 1.56), penetrating injury (OR 2.95), need for at least one other emergency operation (OR 2.09), a “relatively less severe” injury pattern (worst injury is “only” AIS = 3; OR 1.71) and admission on weekend and during daytime (OR 1.28); Conversely, factors significantly oppose Rapid CWR included: higher age (OR 0.99 per year), male sex (OR 0.81) and the presence of other thoracic injuries (hemothorax (OR 0.55), contusion (OR 0.76), laceration (OR 0.68))

The propensity score model for predicting Rapid (Day 0) versus Early (Day 1–3) CWR had modest discrimination (Nagelkerke’s R²=0.115) (Fig. 4), consistent with substantial unmeasured variation in timing decisions. The Odds ratio is shown as a Forest Plot in Fig. 5.

Factors that significantly favored Rapid CWR included:

- Relevant injury besides the thorax (OR 1.56).
- Penetrating injury (OR 2.95).
- Need for at least one other emergency operation (OR 2.09).
- A “relatively less severe” injury pattern (worst injury is “only” AIS = 3; OR 1.71).
- Admission on weekend and during daytime (OR 1.28).

Conversely, factors significantly oppose Rapid CWR included:

- Higher age (OR 0.99 per year).
- Male sex (OR 0.81).
- The presence of other thoracic injuries (hemothorax (OR 0.55), contusion (OR 0.76), laceration (OR 0.68)).

Propensity score matching yielded 500 well-balanced pairs of Rapid and Early CWR patients (*n* = 1,000 total). In the matched cohort, most baseline characteristics were closely aligned:

As shown in Table 3, the age, ISS and gender were nearly equally distributed. And so were specific injuries (thoracic organ injuries i.e. lung laceration 11.6% vs. 9.8%, hemothorax 41.8% vs. 40.8% and lung contusion 40.6% vs. 38.8%). Admission characteristics, the initial treatment in Level 1 Trauma Centers and the admission to the Trauma Resuscitation Unit (TRU) via helicopter transport, additional emergency operations, and early transfusions were comparable.

**Table 3:** Propensity score corresponding to the probability of receiving rapid sequence surgery (Day 0).

Case characteristics	Rapid (day 0) *N* = 500	Early (day 1–3) *N* = 500	SMD
Age	57.8 (18.3)	57.7 (16.3)	0.006
Age over 80 y	63 (12.6%)	46 (9.2%)	0,109
Gender (male)	382 (76.4%)	392 (78.4%)	0,048
ISS	28.1 (12.9)	26.2 (12.1)	0.152
Severe TBI (AIS 4–6)	72 (14.4%)	47 (9.4%)	0,155
Abdominal trauma (AIS 3+)	68 (13.6%)	73 (14.6%)	0,029
Trauma to extremities (AIS 3+)	120 (24.0%)	115 (23.0%)	0,024
Polytrauma (Berlin)	161 (32.2%)	129 (25.8%)	0,141
Penetrating trauma	9 (1.8%)	4 (0.8%)	0,088
Sever Rib fractures (AIS)3 4 5	253 (50.6%) 144 (28.8%) 103 (20.6%)	270 (54.0%) 153 (30.6%) 77 (15.4%)	0,068 0,039 0,136
Lung laceration	58 (11.6%)	49 (9.8%)	0,058
Hemothorax	209 (41.8%)	204 (40.8%)	0,020
Lung contusion	203 (40.6%)	194 (38.8%)	0,037
Lung contusion identified after ICU admission	7 (1.4%)	--	
Admission at night	165 (33.0%)	161 (32.2%)	0,017
Admission during the weekend	208 (41.6%)	209 (41.8%)	0,004
TC Level 1 (versus 2–3)	425 (85.0%)	428 (85.6%)	0,017
Transport by helicopter	129 (26.5%)	145 (30.0%)	0,078
Emergeny surgical procedure	145 (29.0%)	134 (26.8%)	0,049
Blood transfusion (RBC) in TRU/OR	103 (20.6%)	84 (16.8%)	0,098
Base Excess <= -6	83 (18.1)	72 (16.0)	0,056
Coagulopathy	86 (17.8)	63 (13.2)	0,127
Hb < 7	11 (2.2)	3 (0.6)	0,136
**Outcomes**			
Sepsis	63 (14.6%)	51 (12.0%)	
Organ failure lungs	118 (27.1%)	105 (24.1%)	
MOF	147 (33.6%)	123 (28.3%)	
Respirator dependency (days)	1 (0–9) MW 6.5	1 (0–9) MW 6.0	
Length of stay ICU	7 (2–17) MW 11.6	8 (3–16) MW 11.5	
Length of stay hospital	18 (10–29) MW 22.9	17 (11–26) MW 22.7	
Mortality	53 (10.6%)	18 (3.6%)	
RISC III prognosis	16.2%	10.7%	

Matching was performed exactly according to the rounded percentage probability. Propensity score matching yielded 2 × 500 cases (*n* = 1000), with one patient in each pair treated on Day 0 (Rapid) and the other on Days 1–3 (Early). The following table shows the comparative results. Including Standardized mean differences (SMD)

Despite this, some residual differences persisted, indicating incomplete control of pre-existing risk: The ISS remained higher in Rapid vs. Early (28.1 ± 12.9 vs. 26.2 ± 12.1), while severe head injury (AIS_Head_ 4–6) remained more frequent (14.4% vs. 9.4%). Polytrauma (by Berlin criteria) was more common in Rapid (32.2% vs. 25.8%). The RISC III predicted mortality was substantially higher in Rapid (16.2% vs. 10.7%).

Thus, even after matching on the propensity to receive Day 0 CWR, the Rapid group represented a cohort with a higher intrinsic risk of death.

### Outcomes in the matched cohort

In the matched cohort of 500 Rapid and 500 Early CWR patients, the primary outcome - in-hospital mortality - differed significantly: the mortality in the Rapid group was 10.6% (53 of 500) versus 3.6% (18 of 500) in the Early group (*p* < .001).

When compared with the expected mortality based on RISC III, the observed mortality in each group was consistent with the underlying risk: 10.6% observed vs. 16.2% predicted in Rapid, and 3.6% observed vs. 10.7% predicted in Early (the observed mortality is considerably lower here since early deaths were excluded).

Secondary outcomes showed less pronounced differences: Sepsis occurred more often in 14.6% of Rapid versus 12.0% of Early patients (*p*>.271), MOF occurred in 33.6% vs. 28.3% (*p*>.092). The mean ventilation duration was nearly identical (6.5 vs. 6.0 days). The same applies to ICU LOS, which was similar (11.6 vs. 11.5 days on average) as well as hospital LOS, which did not differ materially in the two groups (22.9 vs. 22.7 days).

Thus, aside from mortality, most short-term outcome metrics were comparable between Rapid and Early CWR after matching.

Despite matching, the Rapid group remained more severely injured and at higher baseline risk, with higher ISS, more frequent severe head injury, higher polytrauma frequency, and a substantially worse RISC III prognosis (16.2% vs. 10.7%). This indicates incomplete control of pre-existing differences in injury severity and physiology.

When compared with predicted mortality, both groups performed better than expected: in the Rapid cohort, 10.6% observed versus 16.2% predicted mortality, and in the Early cohort, 3.6% observed versus 10.7% predicted. These findings suggest that CWR, whether performed on Day 0 or Days 1–3, may contribute to improved survival relative to baseline risk.

Standardized mean differences (SMD) for most covariates were reduced to < 0.1 after matching (Table 3), indicating good balance for those variables that entered the propensity model.

### Descriptive outcomes in Delayed and Late CWR groups

For completeness, patients who underwent CWR on Days 4–6 (Delayed; *n* = 360) and Day 7+ (Late; *n* = 385) were also characterized. Compared with Rapid and Early, Late CWR patients showed a slightly higher age (60.8 ± 15.5 years), a higher ISS (29.4 ± 13.0) and more frequent hemothorax and lung contusion. They also showed higher rates of sepsis (21.2%) and MOF (39.8%). The median ventilation time was longer (3 [0–19] days; mean 10.9 days) and they substantially showed a longer ICU LOS (median 15 [4–27] days; mean 19.3 days) as well as prolonged hospital LOS (median 27 [20–42] days; mean 34.4 days). Their mortality rate was with 5.7% in between Rapid and Early.

Given that these groups survived beyond 3 days and initially were managed non-operatively, direct mortality comparisons with Rapid and Early are not appropriate, but their high complication burden seems to underscore the known risks of delayed CWR [1, 2, 11, 17–19].

## Discussion

In this large multicenter registry analysis of 2,498 operatively treated patients with severe chest wall injuries, almost half underwent “rapid sequence” CWR on the day of admission, and nearly a quarter had “early” CWR on days 1–3. Contrary to our original hypothesis, rapid sequence CWR on day 0 was associated with a nearly threefold higher unadjusted in-hospital mortality compared with CWR on days 1–3, a difference that persisted even after propensity score matching. At the same time, both Rapid and Early cohorts experienced mortality rates lower than those predicted by the RISC III model, and they showed very similar rates of sepsis, MOF, ventilator days, and ICU/hospital LOS.

These findings must be interpreted through the lens of confounding by indication and survivorship bias. The decision to perform immediate CWR on day 0 is not randomized, but rather tightly coupled to the overall trauma burden, the presence of life-threatening injuries, and the operative strategy required for hemorrhage and organ injury control. In our propensity model, penetrating trauma, concomitant emergency operations, and transfusion requirement were strong predictors of Day-0-CWR, while “clean” thoracic patterns with contusions and parenchymal injuries actually reduced the likelihood of rapid CWR. Even after matching, patients in the Rapid group had higher ISS, more frequent severe head injury, a higher polytrauma rate, and substantially worse RISC III prognoses than their Early counterparts.

In essence, the Rapid CWR cohort represents a distinct, highly selected population of critically injured patients for whom immediate surgery - including CWR - is deemed essential for survival. By definition, they include unstable patients who might not survive long enough to receive “early” CWR on days 1–3. Conversely, patients in the Early group are survivors of the initial 24- to 72-hour high-risk window, intrinsically enriched for better physiological reserve, fewer devastating extra-thoracic injuries, and favorable response to initial resuscitation [19, 20]. It is therefore unsurprising that, even after matching, the Rapid group carries a higher absolute mortality. Thus, while unadjusted mortality was almost threefold higher in Rapid than Early, both groups actually experienced fewer deaths than predicted, suggesting a beneficial effect of CWR within both timing windows rather than harm from early surgery.

Importantly, the comparison with predicted mortality suggests that this is not evidence that Day-0-CWR per se is harmful. In the matched cohort, Rapid CWR patients had a predicted mortality of 16.2% and observed mortality of 10.6% and Early CWR patients had predicted 10.7% and observed 3.6%. Both represent an absolute survival benefit relative to baseline risk. Our study therefore aligns with, rather than contradicts, the robust literature supporting the benefit of CWR in appropriately selected patients [2, 6, 7, 10, 11, 18, 19]. What our data illustrate is that Day 0 and Day 1–3 cohorts are not interchangeable in terms of case mix, nor should outcomes between them be naively compared without considering pre-injury risk.

### Rapid sequence CWR in context of existing timing literature

Our findings also need to be integrated with the broader timing literature. Multiple studies—including the AAST plenary paper by Pieracci et al. — have shown that SSRF / CWR performed within 72 h is associated with better outcomes than SSRF / CWR performed later [10, 11]. Becker et al., in a matched-pair analysis from the TR-DGU, found that early SSRF / CWR (within 48 h) was associated with reduced pneumonia and ICU LOS compared with later SSRF / CWR, with a possible trend towards reduced mortality [2]. Single-center experiences from Germany and elsewhere have demonstrated that “Day 0–1” CWR can be safely implemented in selected polytrauma patients, often allowing extubation within hours, even in the presence of complex thoracic and extra-thoracic injuries [12, 15, 21].

Our registry-level analysis does not contradict these data. Rather, it refines them by revealing the heterogeneity within the “<72-hour” category. The WSES/CWIS position paper recommends CWR “as early as possible, ideally within 72 hours,” but does not mandate Day 0 CWR for all patients [10]. Our results support this nuanced view: while CWR within 72 h appears beneficial across risk strata, the subset of patients for whom Day 0 CWR is chosen comprise a particularly high-risk group with a mortality profile that reflects the severity of their other injuries more than the effect of CWR timing.

### Confounding by indication and residual bias

Despite rigorous propensity score matching, we were unable to fully equalize the risk profiles of Rapid and Early cohorts, as evidenced by residual differences in ISS, RISC III, and severe head injury rates. This reflects limitations inherent to registry data:

The TR-DGU records injury severity by AIS and ISS but lacks granular anatomical detail, such as the exact location and pattern of each rib fracture (e.g., anterior vs. lateral vs. posterior flail segments) or the distinction between “implosion” injuries and simple series fractures [4, 21]. Physiological trajectories and response to resuscitation are captured only by “snapshot” variables (e.g., base excess, coagulation status) and not by continuous hemodynamic monitoring or lactate clearance. The registry does not differentiate whether CWR was the primary operative indication, an adjunct to another life-saving procedure (e.g., thoracotomy, laparotomy), or performed during a combined “damage control” operation. We had only day-level, not hour-level, timing. A “Day 0” operation could occur within 1 h of arrival or at 23:59, while a “Day 1” operation might occur at 02:00 on the calendar day after a late-evening admission. Thus, there is considerable overlap in actual elapsed time from injury to surgery between Day 0 and Day 1 patients.

Our findings must be interpreted through the lens of confounding by indication and survivorship bias. The decision to perform immediate CWR on Day 0 is tightly coupled to the presence of life-threatening injuries and the perceived need for emergent operative intervention. Penetrating trauma, concomitant emergency operations, and early transfusions were strong predictors of Day-0 CWR in our propensity model, while ‘clean’ thoracic patterns with contusions and parenchymal injuries actually reduced the likelihood of rapid CWR. Even after matching, Rapid patients had higher ISS, more frequent severe head injuries, higher polytrauma rates, and worse RISC III prognoses, underscoring that they constitute a distinct, intrinsically higher-risk population. In exploratory analyses, we also examined outcomes across strata of baseline risk (RISC III) and of the propensity score within each timing group. These descriptive stratifications showed the same qualitative pattern: within each risk stratum, Day-0 patients had higher absolute mortality than Day-1–3 patients, but in both timing groups observed mortality remained below RISC III-predicted values. Because these analyses did not change the interpretation and were limited by small numbers in some strata, we did not include full tables, but they are available on request.

A survivorship bias is also inherent: patients undergoing CWR on Days 1–3 must survive the initial 24–72 h, whereas Day-0 patients include the most unstable individuals who might not have survived for delayed surgery. Excluding deaths within 48 h reduces but does not eliminate this bias. Consequently, the lower mortality observed in the Early group reflects, at least in part, a survivor cohort with better physiological reserve, rather than a protective effect of delayed surgery.

These findings must not be interpreted as evidence that Day-0 CWR is harmful. Instead, they reflect that Day-0 CWR is preferentially used in patients with more severe concomitant injuries and poorer baseline prognosis, in whom immediate operative intervention is part of a life-saving strategy.

The modest explanatory power of our propensity model (Nagelkerke’s R² = 0.115) and the persistence of residual imbalance in ISS, severe head injury, polytrauma rate, and RISC III after matching indicate that the clinical criteria - such as detailed rib fracture morphology, location and extent of flail segments, and dynamic hemodynamic response to resuscitation - leading to Day-0 CWR are heterogeneous and only partially captured by routine registry variables. This heterogeneity is, in itself, an important finding: despite the availability of international consensus statements [10, 22], it remains difficult in everyday practice to operationalize which patients are most likely to benefit from immediate surgery using standard trauma data alone. These structural limitations prevent full equalization of baseline risk between Rapid and Early cohorts and preclude definitive causal inference regarding the effect of timing on mortality.

### Clinical implications

From a clinical perspective, our data argue against a simplistic translation of “earlier is always better” into a blanket policy of operating on all patients with chest wall instability on Day 0. Several important implications emerge:

1. CWR within 72 h appears to be beneficial across a wide range of patients with chest wall instability, in line with existing randomized trials, observational studies, and international guidelines [1, 2, 6, 7, 9–11].
2. The specific subgroup undergoing Day 0 CWR represents a distinct, high-risk population whose outcomes cannot be directly compared to those stabilized on days 1–3. Their higher mortality is primarily attributable to more severe concomitant injuries and early physiological derangement, not necessarily to the timing of CWR.
3. For the majority of hemodynamically stable patients with chest wall instability - especially those without ongoing hemorrhage, refractory shock, or emergent concomitant surgical indications - planning CWR within the first 24–72 h appears appropriate and sufficient. This strategy allows optimization of pain control, respiratory function, and comorbidity management, and accommodates necessary imaging and team coordination [14, 22].
4. Day 0 CWR should remain a tailored, case-by-case decision driven by life-threatening indications (e.g., open chest, major air leak, impaled or penetrating chest wall, hemodynamic instability requiring thoracotomy or laparotomy) rather than a dogmatic target.

Importantly, both Rapid and Early cohorts experienced mortality rates considerably lower than those predicted by RISC III, despite representing high-risk populations. This pattern is consistent with a beneficial contribution of CWR within the broader context of modern chest trauma care. Thus, the higher absolute mortality in the Day-0 cohort should not be interpreted as evidence that immediate CWR is harmful. Rather, it reflects the fact that Day-0 CWR is preferentially used in patients with more severe concomitant injuries and poorer baseline prognosis, in whom CWR is one component of a complex life-saving operative strategy. In patients with severe concomitant injuries or ongoing hemodynamic instability, a ‘rapid sequence’ Day-0 CWR strategy may amplify perioperative risk, particularly when combined with complex emergency procedures and persistent coagulopathy. Anticipated clinical deterioration due to associated injuries or systemic inflammation may sometimes argue against ultrarapid chest wall stabilization and in favor of postponing CWR until the patient has been physiologically stabilized. These considerations reinforce the need for evidence based but individualized strategies.

Within the limitations of this observational registry study, our findings are consistent with the broader literature suggesting benefits of CWR performed within 72 h [10, 22], while underscoring the need for individualized timing decisions that integrate chest wall stability into the overall polytrauma management strategy. However, they caution against extrapolating ‘earlier is better’ into a blanket policy of Day-0 CWR for all patients with chest wall instability. For the majority of hemodynamically stable patients, without ongoing hemorrhage or other emergent indications, planned CWR within 24–72 h allows physiological optimization, comprehensive imaging, and appropriate multidisciplinary planning. Day-0 CWR should remain a tailored, case-by-case decision driven primarily by life-threatening concomitant injuries or mandatory thoracic/abdominal surgery, rather than by an arbitrary time target alone.

### Relation to biomechanical and interventional data

Biomechanical studies have clearly shown that chest wall instability dramatically alters respiratory mechanics and thoracic spine loading, and that rigid fixation with locking plates restores near-normal kinematics [4]. Clinical series have confirmed that CWR, including sternum fixation and transsternal plating, can restore effective breathing, reduce pain, and allow early weaning from mechanical ventilation in both traumatic and CPR-related instability [12, 14, 15]. Our data are congruent with these findings: ventilation days and ICU LOS in our Rapid and Early groups were approximately 6–7 days and 11–12 days, respectively - relatively short durations for patients with a mean ISS in the high 20s, high rates of lung contusion, and frequent extra-thoracic injuries.

In addition, both groups demonstrated mortality rates considerably lower than RISC III predictions, supporting the contention that CWR contributes to improved survival and respiratory outcomes in high-risk chest trauma patients. The fact that this benefit is apparent in both Day 0 and Days 1–3 cohorts underscores that the fundamental question is not whether CWR should be done, but rather how to time and tailor it within the broader polytrauma management algorithm [17, 19, 22].

### Strengths and limitations

This study has several strengths. It is the largest to date focusing specifically on the comparative timing of CWR within the early (< 72-h) window. The data derive from a high-quality, audited national trauma registry with standardized data collection in more than 600 hospitals and > 34,000 patients with severe chest wall injuries. We applied rigorous inclusion and exclusion criteria, and used propensity score matching to address confounding by indication. We benchmarked observed mortality against RISC III predictions, providing important context for interpretability.

Despite the inherent limitations of an observational design, analyses using propensity score methods in this very large, standardized trauma registry arguably represent the closest approximation to ‘prospective quality data’ currently achievable in a retrospective registry setting, and thus provide an important, albeit imperfect, source of evidence.

Limitations include the observational design and lack of randomization. As detailed above, residual confounding by indication and survivorship bias remain substantial despite propensity score matching and exclusion of early deaths. The registry records operation dates but not exact times for rib fixation, which may introduce some misclassification between Day 0 and Day 1 procedures. The absence of hour-level timing and detailed radiologic and physiologic data limits our ability to refine exposure classification and risk adjustment. Approximately one quarter of operative CWR cases were excluded due to missing surgery date, which may introduce selection bias. Although we have no evidence that their inclusion would systematically change effect estimates, this missingness could introduce selection bias. The propensity model had modest explanatory power, and the registry lacks granular anatomical detail (e.g., fracture location, flail segment type) and detailed physiological trajectories, preventing precise characterization of all factors influencing timing decisions. Long-term functional and quality-of-life outcomes, which are critical given the lasting impact of rib fractures, were not available [23, 24].

## Conclusion

In this large, propensity score-matched registry analysis of patients with severe chest wall injuries undergoing CWR, rapid sequence CWR on the day of admission was associated with significantly higher in-hospital mortality compared to CWR performed on Days 1–3, despite similar short-term morbidity profiles. However, patients selected for Day-0-CWR had a higher baseline injury burden and worse predicted mortality even after matching, and both Rapid and Early cohorts exhibited mortality rates lower than those predicted by RISC-III-Score.

These findings suggest that the higher mortality among Day-0 patients reflects residual confounding by indication and the intrinsically higher risk profile of patients selected for immediate surgery, rather than a harmful effect of rapid CWR itself.

Within the limitations of this observational registry study, our findings are consistent with the broader literature suggesting benefits of CWR performed within 72 h, while underscoring the need for individualized timing decisions that integrate chest wall stability into the overall polytrauma management strategy: when CWR is indicated, current evidence and our findings suggest that performing it within the first 72 h is desirable whenever it can be done safely. However, in hemodynamically unstable patients or those with critical associated injuries, the risks of immediate surgery may outweigh the potential benefits of very early stabilization, and timing must be individualized. The low explanatory power of our propensity model and the heterogeneity of timing determinants underscore the need for prospective studies with detailed imaging and physiological data to better identify those patients who truly benefit from immediate versus early (Day-1–3) CWR.

## Data Availability

Data sharing not applicable to this article as no datasets other than from the TraumaRegister DGU^®^ were generated or analysed during the current study. On reasonable request, subject to institutional and ethical approvals, anonymized data and analytical code are available from the corresponding author.
